# Epidemiological Research on Occupational and Environmental Carcinogens

**DOI:** 10.3390/ijerph18052215

**Published:** 2021-02-24

**Authors:** Caterina Ledda

**Affiliations:** Occupational Medicine, Department of Clinical and Experimental Medicine, University of Catania, 95100 Catania, Italy; cledda@unict.it

**Keywords:** cancer, epidemiology, prevention, predictive, precision, medicine, exposure, biomarkers, vulnerable

## 1. Introduction

The cancer risk associated with exposure to environmental and occupational carcinogens such as asbestos, benzene, radiation, or lifestyle carcinogens such as cigarette smoking depends on the entire history of exposure to the carcinogen, including the age of exposure and the time-varying intensity of exposure. While the importance of temporal aspects of exposure and risk has been highlighted for some time, the vast majority of epidemiological studies of cohorts use cumulative exposure as the measure of exposure, even when detailed exposure data are available for each member of the cohort. One reason is that the detailed history of exposure is difficult to incorporate in the traditional statistical models used to analyze epidemiological data. In contrast, time-dependent parameters associated with time-varying concentrations of exposure can be easily accommodated in statistical analyses based on multistage models.

Principally, as a result of the focus on case-control studies, undue emphasis has been placed on the development of relative risk regression models. Even when cohort data are accessible, epidemiological studies have focused on estimating relative risk when the more appropriate targets of estimation are the hazard functions for varying levels of exposure. After the hazard functions are estimated, numerous procedures of risk, such as relative risk and excess risk, can be simply estimated. Methods of analyses constructed on multistage models provide one approach to estimating hazard functions for any general time-varying exposure history.

Finally, multistage models for carcinogenesis provide a combined framework for analyses of data from multiple sources in cancer epidemiology.

## 2. Epidemiological Research on Occupational Carcinogens

Bernardino Ramazzini (1633–1714), the father of occupational medicine, studied several workers’ diseases in 52 professions and reported and introduced for the first time the concept of occupational cancers [1].

Occupational cancer epidemiology has advanced from identifying high relative risks for rare cancers in definite worker groups, such as Percival Pott’s discovery, to noticing moderate associations among carcinogens and more common cancers [1]. Although epidemiological studies of occupational cancer have expanded since the 1950s, specific organizational topics warrant more attention [2]. Primarily, occupational cancer epidemiology studies are infrequently comprised of women and minority study subjects [3,4]. These investigations can also have inadequate industrial hygiene information, useful for clarifying exposure–response associations [5]. While research approaches have upgraded and data source accessibility has increased [6,7], there is a sense that the level of effort focused on occupational cancer investigation may have declined in recent years [5,8].

Recently, the “European Agency for Safety & Health at Work” reported that worldwide occupational cancers reached approximately 9.6% of all cancer deaths [9]. 

Organizations such as the International Agency for Research on Cancer (IARC), the United States Environmental Protection Agency (EPA), the American Conference of Governmental Industrial Hygienists (ACGIH), and the National Toxicology Program (NTP) want solid epidemiological evidence to elevate or reduce their estimations of carcinogenicity. Unevenly, one-third of definite human carcinogens, as classified by IARC, are work-related exposures [6,10,11]. Several further occupational contacts are recognized as “potentially carcinogenic” and have not been conclusively considered carcinogens because of questionable epidemiological indication or human data absence [10]. Agents presently registered as “potentially carcinogenic” in IARC monographs and the NTP Report on Carcinogens include commonly used pesticides, chemicals, and metals. Besides, certain occupations or industries are associated with cancer, such as meat workers and lung cancer [5,8]. Still, more investigation is essential to recognize the specific agents responsible within the occupation or industry. It is essential to elucidate potential carcinogens’ true nature and generally define suspect occupational groups and industries. A decrease in occupational cancer epidemiology studies could similarly give the incorrect impression that most work-related carcinogens have been recognized and are sufficiently controlled in the working setting [5,8,12].

Recent studies have highlighted how other risk factors such as shift work, night work, and work-related stress are associated with cancer initiation [13,14].

## 3. Epidemiological Research on Environmental Carcinogens

“Environment” is well-defined by the World Health Organization (WHO) for the determination of environmental attribution as “all the physical, chemical and biological factors peripheral to the human host, and all related behaviors, but excluding those natural environments that cannot reasonably be modified” [15,16]. This classification is partial to those parts of the environment that can, in theory, be improved to decrease the impact of the environment on health. It also ignores those behaviors and lifestyles not strictly connected to environmental exposures, such as alcohol consumption and tobacco use, and behaviors associated with the social and cultural environment, genetics, and parts of the “unmodifiable” natural environment [15,16].

People are exposed to several carcinogenic agents through inhalation, eating, drinking, and skin contact. Meanwhile, since most people work for closely two-thirds of their time, they have many, and frequently prolonged, chances for connections with occupational carcinogens, consequential in the accumulation of exposure over a lifetime. WHO has estimated that a substantial proportion of all cancers are attributable to the environment [16,17]. Environmental factors that upsurge risks for emerging cancer characteristically affect the overall population through spontaneous exposures, over which persons have little control. Exposure to most carcinogens is inclined to be most significant in the most underprivileged sections of the individuals [18,19].

## 4. Future Perspectives

Epidemiology helps to understand the distribution of cancer in people and the causes, consequences, prevention, and treatment strategies. The need to couple epidemiological studies observing epigenetic damage is increasing [20,21,22].

Epigenetics is well-defined as heritable changes in gene expression that occur without changes to the underlying DNA sequence. Types of epigenetic regulators comprise DNA methylation, histone modifications, microRNA, and prions [20,23].

Epigenetics has been welcomed as a missing mechanistic connection between environmental and occupational exposures or genetics and many common diseases [20,24,25,26].

In addition, investigation of high-throughput genomic data is inspiring and necessitates specialized knowledge of experimental design, genomic data preprocessing and quality control, high-dimensional data analysis, and machine learning [27,28,29,30].

It is, therefore, essential to focus scientific activity on knowledge between cancer and the environment and work, in order to carry out the right monitoring and prevention activities [31,32].

## Data Availability

Not applicable.
